# Supplementary material for: Yeast Diversity in the Qaidam Basin Desert in China with the Description of Five New Yeast Species
Source: J Fungi (Basel). 2022 Aug 16;8(8):858. doi: 10.3390/jof8080858 (PMC9409814; doi:10.3390/jof8080858)
Supplement: Supplementary file 1 [file jof-08-00858-s001.zip › Supplementary files_Figure S1-S2.pdf]

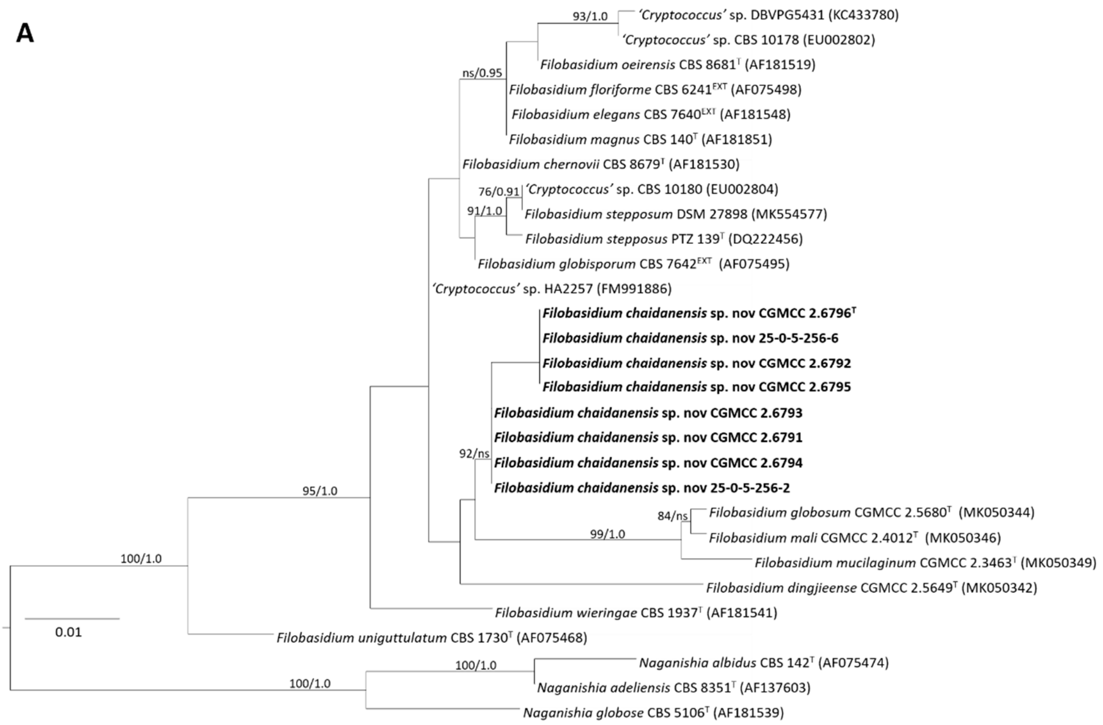
A
93/1.0
'Cryptococcus' sp. DBVPG5431 (KC433780)
'Cryptococcus' sp. CBS 10178 (EU002802)
Filobasidium oeirensis CBS 8681T (AF181519)
ns/0.95
Filobasidium floriforme CBS 6241EXT (AF075498)
Filobasidium elegans CBS 7640LXT (AF181548)
Filobasidium magnus CBS 140T (AF181851)
Filobasidium chernovii CBS 8679T (AF181530)
76/0.91
'Cryptococcus' sp. CBS 10180 (EU002804)
91/1.0
Filobasidium stepposum DSM 27898 (MK554577)
Filobasidium stepposus PTZ 139T (DQ222456)
Filobasidium globisporum CBS 7642EXT (AF075495)
'Cryptococcus' sp. HA2257 (FM991886)
Filobasidium chaidanensis sp. nov CGMCC 2.6796T
Filobasidium chaidanensis sp. nov 25-0-5-256-6
Filobasidium chaidanensis sp. nov CGMCC 2.6792
Filobasidium chaidanensis sp. nov CGMCC 2.6795
Filobasidium chaidanensis sp. nov CGMCC 2.6793
Filobasidium chaidanensis sp. nov CGMCC 2.6791
92/ns
Filobasidium chaidanensis sp. nov CGMCC 2.6794
Filobasidium chaidanensis sp. nov 25-0-5-256-2
95/1.0
84/ns
Filobasidium globosum CGMCC 2.5680T (MK050344)
Filobasidium mali CGMCC 2.4012T (MK050346)
99/1.0
100/1.0
Filobasidium mucilaginum CGMCC 2.3463T (MK050349)
Filobasidium dingjieense CGMCC 2.5649T (MK050342)
Filobasidium wieringae CBS 1937T (AF181541)
0.01
Filobasidium uniguttulatum CBS 1730T (AF075468)
100/1.0
Naganishia albidus CBS 142T (AF075474)
100/1.0
Naganishia adeliensis CBS 8351T (AF137603)
Naganishia globose CBS 5106T (AF181539)

B

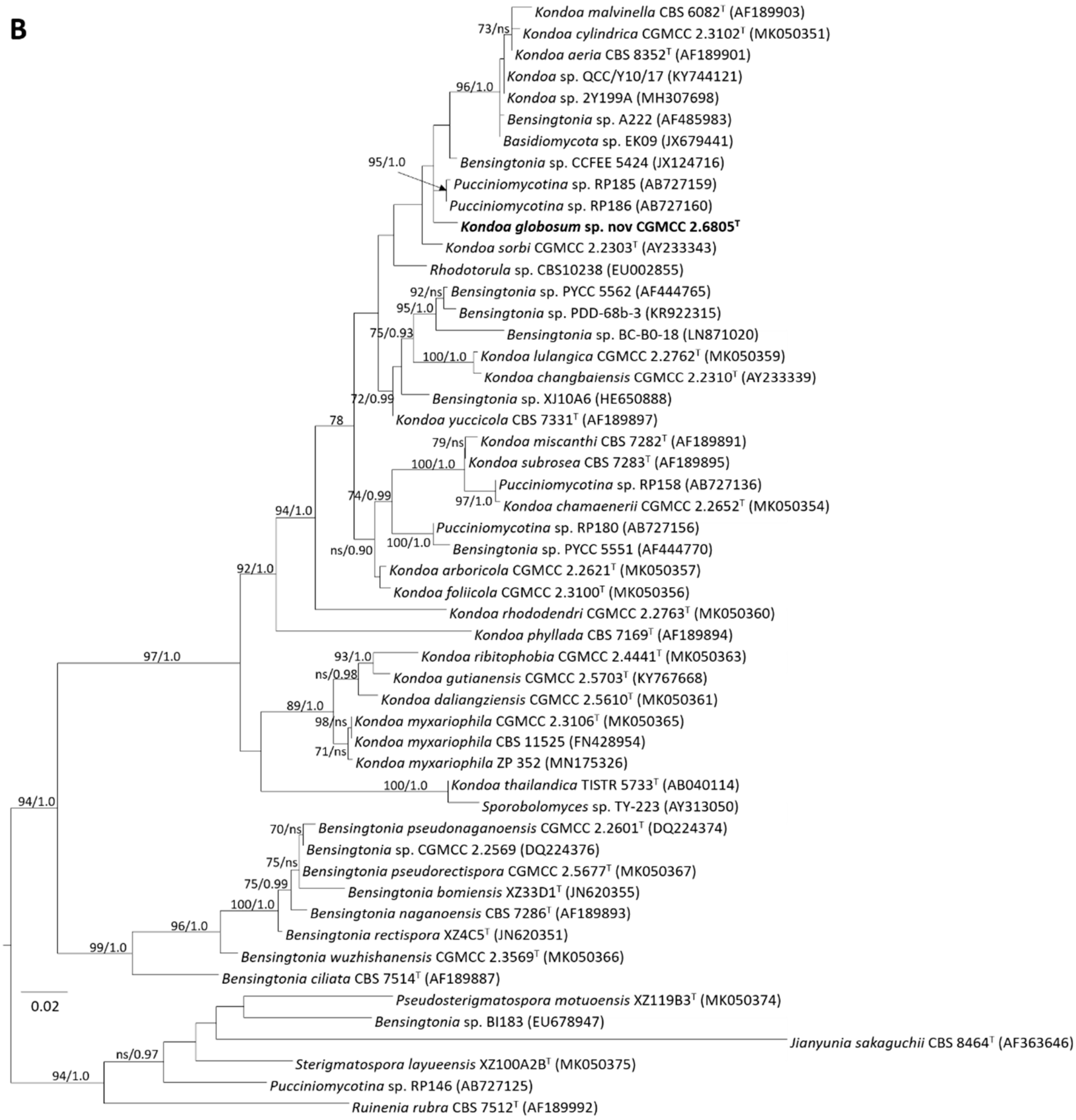
Kondoa malvinella CBS 6082T (AF189903)
73/ns
Kondoa cylindrica CGMCC 2.3102T (MK050351)
Kondoa aeria CBS 8352T (AF189901)
96/1.0
Kondoa sp. QCC/Y10/17 (KY744121)
Kondoa sp. 2Y199A (MH307698)
Bensingtonia sp. A222 (AF485983)
Basidiomycota sp. EK09 (JX679441)
Bensingtonia sp. CCFEE 5424 (JX124716)
95/1.0
Pucciniomycotina sp. RP185 (AB727159)
Pucciniomycotina sp. RP186 (AB727160)
Kondoa globosum sp. nov CGMCC 2.6805T
Kondoa sorbi CGMCC 2.2303T (AY233343)
Rhodotorula sp. CBS10238 (EU002855)
92/ns
Bensingtonia sp. PYCC 5562 (AF444765)
95/1.0
Bensingtonia sp. PDD-68b-3 (KR922315)
Bensingtonia sp. BC-B0-18 (LN871020)
75/0.93
100/1.0
Kondoa lulangica CGMCC 2.2762T (MK050359)
Kondoa changbaiensis CGMCC 2.2310T (AY233339)
Bensingtonia sp. XJ10A6 (HE650888)
72/0.99
Kondoa yuccicola CBS 7331T (AF189897)
78
79/ns
Kondoa miscanthi CBS 7282T (AF189891)
100/1.0
Kondoa subrosea CBS 7283T (AF189895)
Pucciniomycotina sp. RP158 (AB727136)
74/0.99
97/1.0
Kondoa chamaenerii CGMCC 2.2652T (MK050354)
94/1.0
Pucciniomycotina sp. RP180 (AB727156)
100/1.0
ns/0.90
Bensingtonia sp. PYCC 5551 (AF444770)
92/1.0
Kondoa arboricola CGMCC 2.2621T (MK050357)
Kondoa foliicola CGMCC 2.3100T (MK050356)
Kondoa rhododendri CGMCC 2.2763T (MK050360)
Kondoa phyllada CBS 7169T (AF189894)
97/1.0
93/1.0
Kondoa ribitophobia CGMCC 2.4441T (MK050363)
ns/0.98
Kondoa gutianensis CGMCC 2.5703T (KY767668)
Kondoa daliangziensis CGMCC 2.5610T (MK050361)
89/1.0
98/ns
Kondoa myxariophila CGMCC 2.3106T (MK050365)
Kondoa myxariophila CBS 11525 (FN428954)
71/ns
Kondoa myxariophila ZP 352 (MN175326)
100/1.0
Kondoa thailandica TISTR 5733T (AB040114)
94/1.0
Sporobolomyces sp. TY-223 (AY313050)
70/ns
Bensingtonia pseudonaganoensis CGMCC 2.2601T (DQ224374)
Bensingtonia sp. CGMCC 2.2569 (DQ224376)
75/ns
Bensingtonia pseudorectispora CGMCC 2.5677T (MK050367)
75/0.99
Bensingtonia bomiensis XZ33D1T (JN620355)
100/1.0
Bensingtonia naganoensis CBS 7286T (AF189893)
96/1.0
Bensingtonia rectispora XZ4C5T (JN620351)
99/1.0
Bensingtonia wuzhishanensis CGMCC 2.3569T (MK050366)
Bensingtonia ciliata CBS 7514T (AF189887)
0.02
Pseudosterigmatospora motuoensis XZ119B3T (MK050374)
Bensingtonia sp. BI183 (EU678947)
Jianyunia sakaguchii CBS 8464T (AF363646)
ns/0.97
Sterigmatospora layueensis XZ100A2BT (MK050375)
94/1.0
Pucciniomycotina sp. RP146 (AB727125)
Ruinenia rubra CBS 7512T (AF189992)

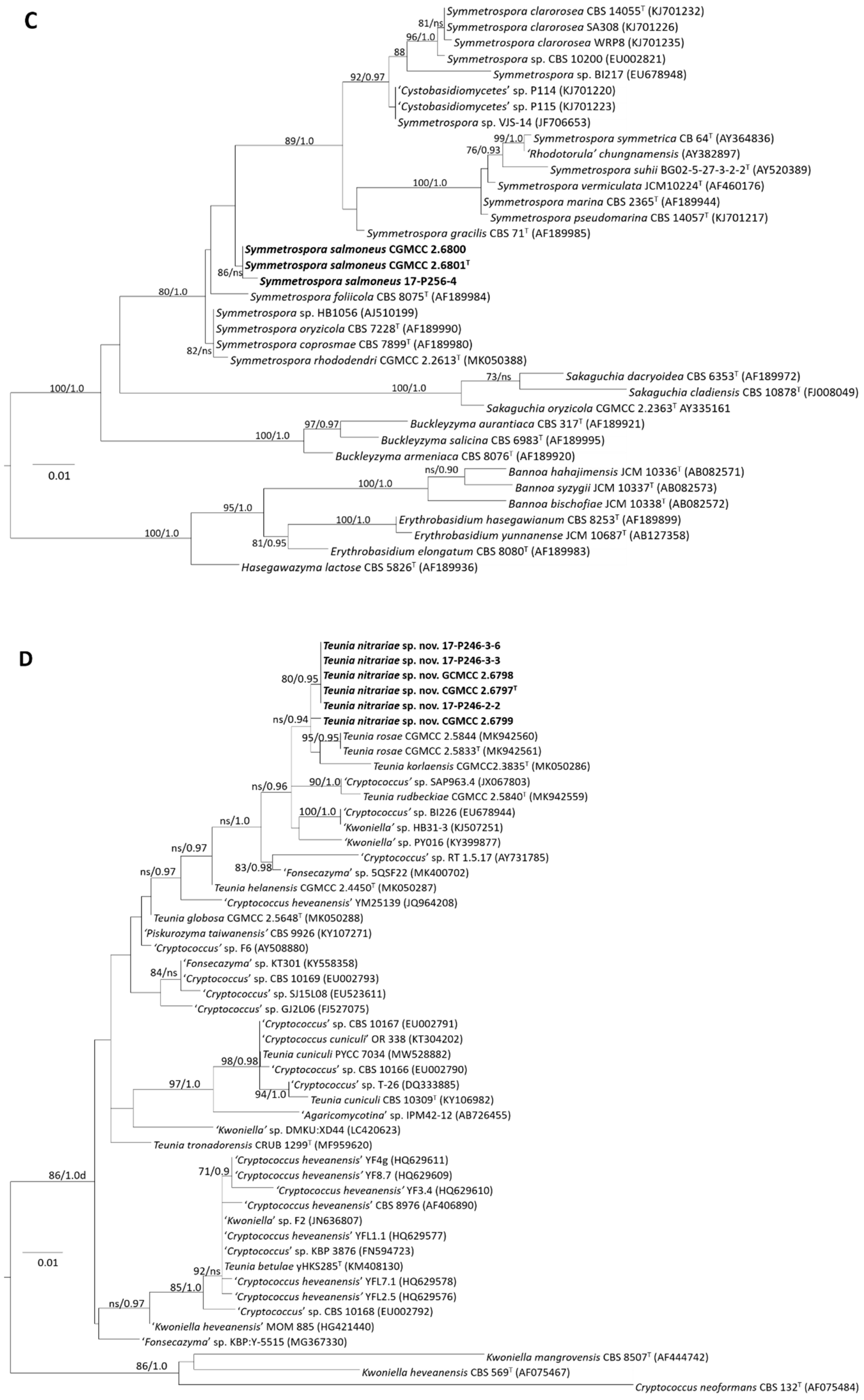

C
Symmetrospora clarorosea CBS 14055T (KJ701232)
Symmetrospora clarorosea SA308 (KJ701226)
Symmetrospora clarorosea WRP8 (KJ701235)
Symmetrospora sp. CBS 10200 (EU002821)
Symmetrospora sp. BI217 (EU678948)
'Cystobasidiomycetes' sp. P114 (KJ701220)
'Cystobasidiomycetes' sp. P115 (KJ701223)
Symmetrospora sp. VJS-14 (JF706653)
Symmetrospora symmetrica CB 64T (AY364836)
'Rhodotorula' chungnamensis (AY382897)
Symmetrospora suhii BG02-5-27-3-2-2T (AY520389)
Symmetrospora vermiculata JCM10224T (AF460176)
Symmetrospora marina CBS 2365T (AF189944)
Symmetrospora pseudomarina CBS 14057T (KJ701217)
Symmetrospora gracilis CBS 71T (AF189985)
Symmetrospora salmoneus CGMCC 2.6800
Symmetrospora salmoneus CGMCC 2.6801T
Symmetrospora salmoneus 17-P256-4
Symmetrospora foliicola CBS 8075T (AF189984)
Symmetrospora sp. HB1056 (AJ510199)
Symmetrospora oryzicola CBS 7228T (AF189990)
Symmetrospora coprosmae CBS 7899T (AF189980)
Symmetrospora rhododendri CGMCC 2.2613T (MK050388)
Sakaguchia dacryoidea CBS 6353T (AF189972)
Sakaguchia cladiensis CBS 10878T (FJ008049)
Sakaguchia oryzicola CGMCC 2.2363T AY335161
Buckleyzyma aurantiaca CBS 317T (AF189921)
Buckleyzyma salicina CBS 6983T (AF189995)
Buckleyzyma armeniaca CBS 8076T (AF189920)
Bannoa hahajimensis JCM 10336T (AB082571)
Bannoa syzygii JCM 10337T (AB082573)
Bannoa bischofiae JCM 10338T (AB082572)
Erythrobasidium hasegawianum CBS 8253T (AF189899)
Erythrobasidium yunnanense JCM 10687T (AB127358)
Erythrobasidium elongatum CBS 8080T (AF189983)
Hasegawazyma lactose CBS 5826T (AF189936)
81/ns
96/1.0
88
92/0.97
89/1.0
99/1.0
76/0.93
100/1.0
86/ns
80/1.0
82/ns
100/1.0
73/ns
100/1.0
97/0.97
100/1.0
ns/0.90
100/1.0
95/1.0
100/1.0
81/0.95
100/1.0
0.01
D
Teunia nitrariae sp. nov. 17-P246-3-6
Teunia nitrariae sp. nov. 17-P246-3-3
Teunia nitrariae sp. nov. GCMCC 2.6798
Teunia nitrariae sp. nov. CGMCC 2.6797T
Teunia nitrariae sp. nov. 17-P246-2-2
Teunia nitrariae sp. nov. CGMCC 2.6799
Teunia rosae CGMCC 2.5844 (MK942560)
Teunia rosae CGMCC 2.5833T (MK942561)
Teunia korlaensis CGMCC2.3835T (MK050286)
'Cryptococcus' sp. SAP963.4 (JX067803)
Teunia rudbeckiae CGMCC 2.5840T (MK942559)
'Cryptococcus' sp. BI226 (EU678944)
'Kwoniella' sp. HB31-3 (KJ507251)
'Kwoniella' sp. PY016 (KY399877)
'Cryptococcus' sp. RT 1.5.17 (AY731785)
'Fonsecazyma' sp. 5QSF22 (MK400702)
Teunia helanensis CGMCC 2.4450T (MK050287)
'Cryptococcus heveanensis' YM25139 (JQ964208)
Teunia globosa CGMCC 2.5648T (MK050288)
'Piskurozyma taiwanensis' CBS 9926 (KY107271)
'Cryptococcus' sp. F6 (AY508880)
'Fonsecazyma' sp. KT301 (KY558358)
'Cryptococcus' sp. CBS 10169 (EU002793)
'Cryptococcus' sp. SJ15L08 (EU523611)
'Cryptococcus' sp. GJ2L06 (FJ527075)
'Cryptococcus' sp. CBS 10167 (EU002791)
'Cryptococcus cuniculi' OR 338 (KT304202)
Teunia cuniculi PYCC 7034 (MW528882)
'Cryptococcus' sp. CBS 10166 (EU002790)
'Cryptococcus' sp. T-26 (DQ333885)
Teunia cuniculi CBS 10309T (KY106982)
'Agaricomycotina' sp. IPM42-12 (AB726455)
'Kwoniella' sp. DMKU:XD44 (LC420623)
Teunia tronadorensis CRUB 1299T (MF959620)
'Cryptococcus heveanensis' YF4g (HQ629611)
'Cryptococcus heveanensis' YF8.7 (HQ629609)
'Cryptococcus heveanensis' YF3.4 (HQ629610)
'Cryptococcus heveanensis' CBS 8976 (AF406890)
'Kwoniella' sp. F2 (JN636807)
'Cryptococcus heveanensis' YFL1.1 (HQ629577)
'Cryptococcus' sp. KBP 3876 (FN594723)
Teunia betulae γHKS285T (KM408130)
'Cryptococcus heveanensis' YFL7.1 (HQ629578)
'Cryptococcus heveanensis' YFL2.5 (HQ629576)
'Cryptococcus' sp. CBS 10168 (EU002792)
'Kwoniella heveanensis' MOM 885 (HG421440)
'Fonsecazyma' sp. KBP:Y-5515 (MG367330)
Kwoniella mangrovensis CBS 8507T (AF444742)
Kwoniella heveanensis CBS 569T (AF075467)
Cryptococcus neoformans CBS 132T (AF075484)
80/0.95
ns/0.94
95/0.95
ns/0.96
90/1.0
100/1.0
ns/1.0
ns/0.97
83/0.98
ns/0.97
84/ns
98/0.98
97/1.0
94/1.0
86/1.0d
71/0.9
92/ns
85/1.0
ns/0.97
86/1.0
0.01

E

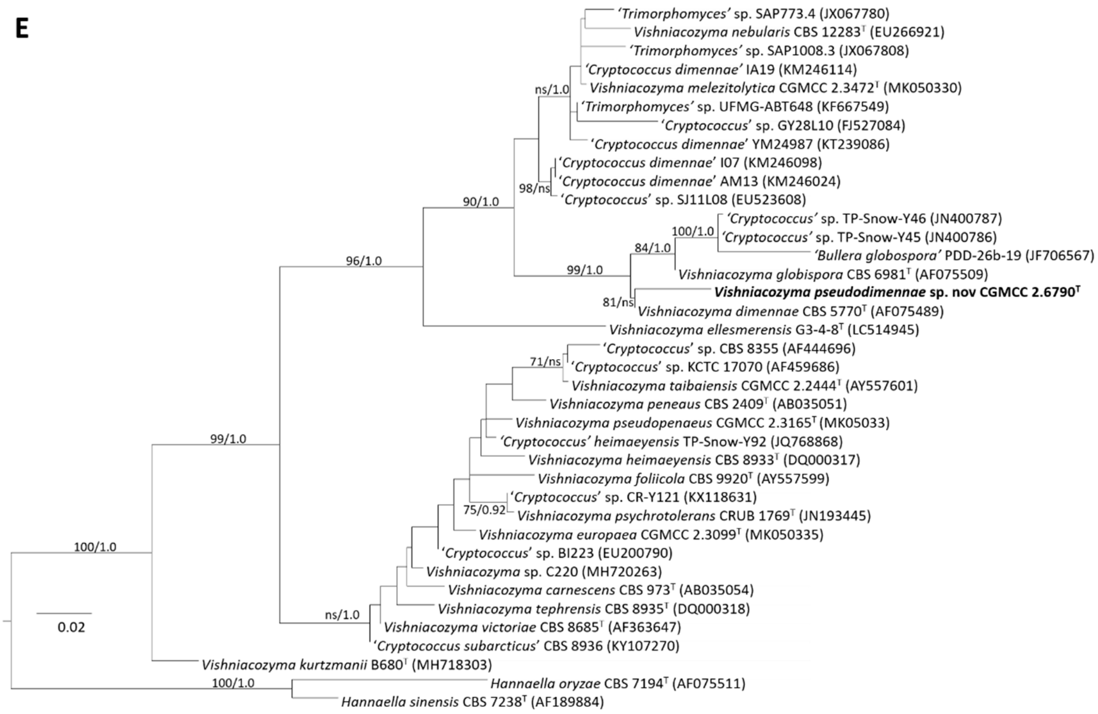

Figure S1. The Maximum likelihood tree of five new yeast species based on D1D2 sequence datasets: (A) *Filobasidium*; (B) *Kondoa*; (C) *Symmetrospora*; (D) *Teunia*; (E) *Vishniacozyma*. The tree backbone is constructed using RAxML and the numbers in each node represent bootstrap percentages (BP) of maximum likelihood from 1000 replicates and Bayesian posterior probabilities (PP). BP values ≥ 70 and PP values ≥ 0.9 were plotted on the branches of the tree. The clade of new species accepted in this study are in bold clades. Scale in 0.02 substitution per nucleotide position.

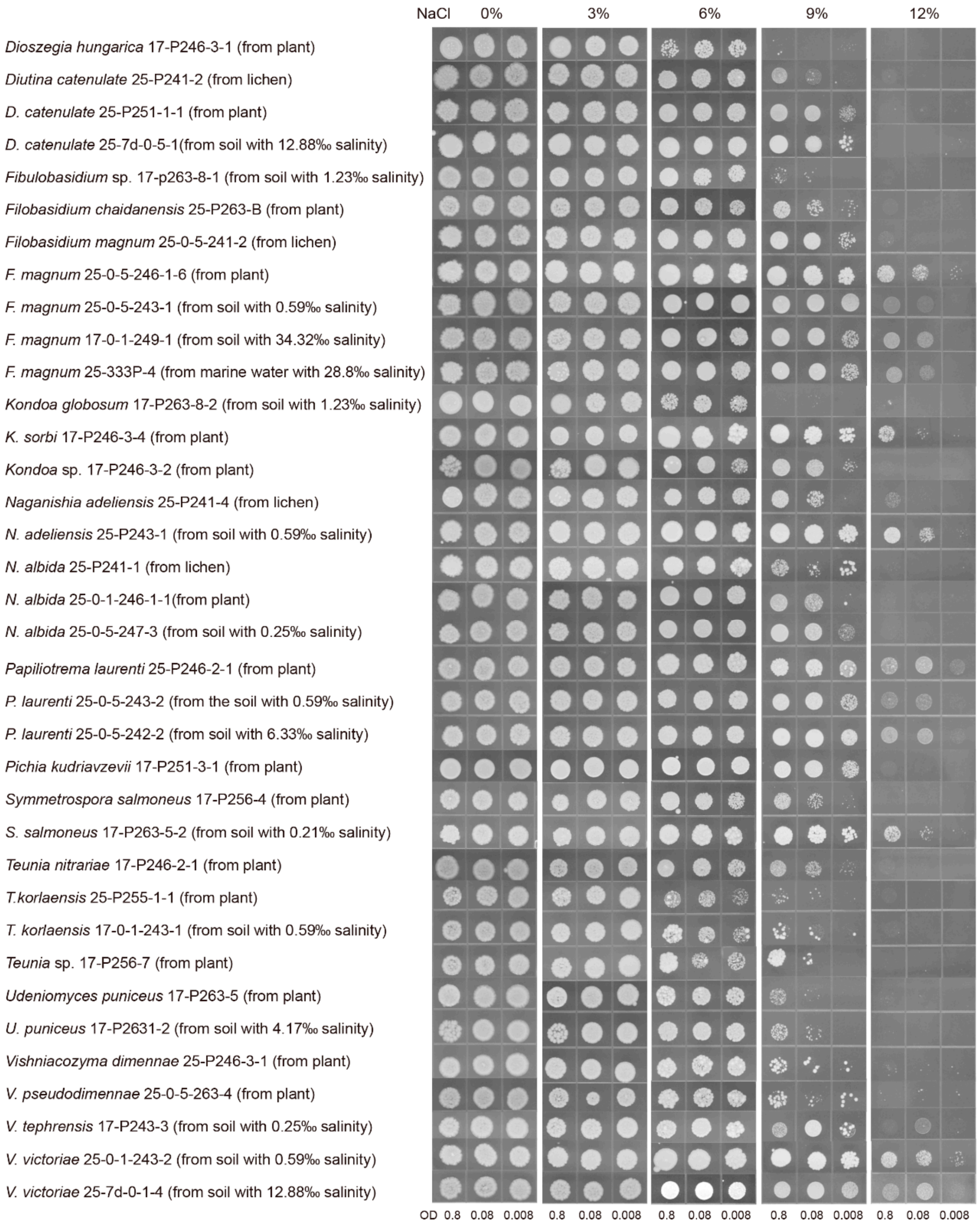

Figure S2. Growth of representative strains of all the species found in this study on YPD plates supplemented with 0%, 3%, 6%, 9%, and 12% (w/v) NaCl. All plates were incubated for 5 days at 20 °C.
